# FungiFun3: systemic gene set enrichment analysis for fungal species

**DOI:** 10.1093/bioinformatics/btae620

**Published:** 2024-11-22

**Authors:** Albert Garcia Lopez, Daniela Albrecht-Eckardt, Gianni Panagiotou, Sascha Schäuble

**Affiliations:** Department of Microbiome Dynamics, Leibniz Institute for Natural Product Research and Infection Biology – Hans-Knöll-Institute (Leibniz-HKI), 07745 Jena, Germany; Biocontrol Jena GmbH, 07745 Jena, Germany; Department of Microbiome Dynamics, Leibniz Institute for Natural Product Research and Infection Biology – Hans-Knöll-Institute (Leibniz-HKI), 07745 Jena, Germany; Institute of Microbiology, Faculty of Biological Sciences, Faculty of Medicine, Friedrich Schiller University Jena Jena, 07743, Germany; Department of Microbiome Dynamics, Leibniz Institute for Natural Product Research and Infection Biology – Hans-Knöll-Institute (Leibniz-HKI), 07745 Jena, Germany

## Abstract

**Summary:**

The ever-growing amount of genome-wide omics data paved the way for solving life science problems in a data-driven manner. Among others, enrichment analysis is part of the standard analysis arsenal to determine systemic signals in any given transcriptomic or proteomic data. Only a part of the members of the fungal kingdom, however, can be analyzed via public web applications, despite the global rise of fungal pathogens and their increasing resistance to antimycotics. We present FungiFun3, a major update of our user-friendly gene set enrichment web application dedicated to fungi. FungiFun3 was rebuilt from scratch to support a modern and easy-to-use web interface and supports >4-fold more fungal strains (*n* = 1287 in total) than its predecessor. In addition, it also allows ranked gene set enrichment analysis at the genomic scale. FungiFun3 thus serves as a starting hub for identifying molecular signals in omics datasets related to a vast amount of available fungal strains including human fungal pathogens of the WHO’s priority list and far beyond.

**Availability and implementation:**

FungiFun3, including sample data and FAQ, is freely available at https://fungifun3.hki-jena.de/.

## 1 Introduction

In October 2022 the World Health Organization (WHO) published a priority list of 19 human fungal pathogens (https://www.who.int/publications/i/item/9789240060241) including, but not limited to, *Candida albicans* and *Aspergillus fumigatus*, which highlights the need to strengthen our efforts to combat fungal infectious diseases (Fisher and Denning 2023). These range from superficial infections to life-threatening systemic diseases, causing great harm to public health and the economy. A pivotal step towards better understanding and exploiting fungal biology towards effective novel therapeutic strategies is meticulous scrutiny of fungal molecular functions. Due to routinely applied high-throughput technologies nowadays, unprecedented amounts of gene expression and protein abundance data offer an opportunity to dissect the genetic underpinnings of fungal infection of humans, plants, or animals in the face of increasingly emerging antifungal resistance (Lockhart *et al.* 2023).

A common technique to systematically investigate genome-wide gene expression or protein abundance data is gene set enrichment analysis, which is typically conducted by querying a list of gene symbols of interest and detecting statistically relevant signals of molecular function. Although results are dependent on the actuality of database annotations and typically ignore complex gene-gene regulatory interaction information, gene set enrichment offers a variety of advantages over single gene expression analysis. Enrichment analysis allows to identify higher-level functional patterns of e.g. differentially expressed gene sets, which can be cumbersome to interpret on their own. It thus contributes to hypothesis generation and testing of molecular regulatory or metabolic pathways, and can be compared across different datasets offering the opportunity to reveal shared signals across multiple tested conditions or different diseases. Gene set enrichment analysis can be done in two ways: (i) by running overrepresentation analysis (ORA) using a list of all differentially expressed genes (optionally filtered for up- or downregulated genes) derived from a comparison of interest (e.g. treatment versus control) or (ii) by adding activity information between two conditions of interest to the query (e.g. sorted expression fold changes for genes associated to the compared conditions) to run a rank-based gene set enrichment analysis (GSEA) (Subramanian *et al.* 2005) against a knowledge database (Huang *et al.* 2009, Maleki *et al.* 2020). A number of tools and annotations exist to run gene set analysis for human, plant, or bacterial organisms (Kuleshov *et al.* 2016, Ge *et al.* 2020, Ma *et al.* 2022, Szklarczyk *et al.* 2023). Investigating the molecular function of fungal species, including fungi not on the top priority list of the WHO as of 2024, however, is hampered by a lack of easy-to-use and readily available tools. Especially, for rare, or not yet supported, fungi, gene set analysis is challenging to achieve, given the need for generating and curating reference genomes, appropriate annotations and molecular function compatible gene sets. We presented FungiFun and its successor FungiFun2 in the past to fill this gap (Priebe *et al.* 2011, 2015). FungiFun2 allowed easy ORA-based analysis of nearly 300 fungal strains using a web interface built upon a mixture of multiple web technologies including PHP and javascript. While comprehensive and up-to-date at the time, both its technological backend and frontend as well as available fungal strains became outdated and limited further usage.

We here present FungiFun3, a major update of fungal gene set enrichment analysis. FungiFun3 was rebuilt completely from scratch as an R Shiny web app. Consolidating the technological framework to R substantially improved its maintainability and enables continuous updates of available fungal strains. The number of supported annotations, including also the human and mouse host systems, increased >4-fold to a total of 1287 annotations at the time of this publication and is continuously being updated. GSEA and additional visualization options including ranked barcode, network, and upset plots were added to allow a focused investigation of the molecular function of fungi as well as the human or mouse host systems. The ease-of-use was further improved and includes example inputs for each available analysis format. The visual output is readily downloadable in publication quality in multiple common formats. Finally, all results are available in spreadsheet formats. Both, accessibility and analysis options pave the way for subsequent analyses of fungal molecular function of both well- and less well-studied fungi. FungiFun3 will thus contribute to identifying promising avenues for studying and understanding emerging fungal resistance to fungicides and antimycotics and thus help to identify novel druggable targets (Du *et al.* 2020, Fisher *et al.* 2022, Lockhart *et al.* 2023).

## 2 Materials and methods

FungiFun3, as opposed to its predecessor, is developed as an R shiny web application. All available genomic annotations are included within a MySQL database using R scripts to allow automated updates while requiring minimal backend activity to update data resources. The supported fungal genomic annotations include categories derived from Gene Ontology (GO), Kyoto Encyclopedia of Genes and Genomes (KEGG), and Functional Catalogue (FunCat, Supplementary Table S1). For GO we collected annotation data from multiple sources including the European Bioinformatics Institute (EBI) (Huntley *et al.* 2015), the FungiDB database (Basenko *et al.* 2018), and UniProt (Bateman *et al.* 2023). In addition, KEGG gene-to-pathway annotations were retrieved using the KEGG REST API (https://rest.kegg.jp/link/pathway/) (Kanehisa *et al.* 2023). The support for the functional annotation scheme FunCat (Ruepp *et al.* 2004) is switched to legacy support with the latest update made in 2015, as both the FunCat resource website and the associated PEDANT database (Walter *et al.* 2009) with hosted FunCat annotations are offline and thus inaccessible at the time of this publication. In summary, 1059 annotations were retrieved from EBI, 289 from FungiDB, 437 from UniProt, and 172 from KEGG, while 177 FunCat categories were kept from FungiFun2 as legacy support. In total, FungiFun3 supports interrogation of 1287 strains (1285 fungal, and 2 two potential host systems, human and mouse).

Based on the R statistical programming language (v4.3.0), several R scripts were combined for data processing of input data, data analysis, and web application deployment as R Shiny App. MySQL retrieval is realized by using the R packages DBI (v1.1.3) and RMySQL (v0.10.25). ORA-based analysis of unranked gene lists is supported by providing Fisher-exact tests or is based on hypergeometric testing. The FGSEA package (v1.28.0) is used to support GSEA enrichment analysis using a ranked input list of all genes (two columns for IDs and ranks) (Korotkevich *et al.* 2021). Parameters for GSEA are further explained in the FAQs section of FungiFun3. Multiple-test correction options including Bonferroni, Benjamini–Hochberg and Benjamini–Yekutieli are available for both ORA and GSEA. Visualizations of resulting enriched categories are provided within the R Shiny App using ggplot2 (v3.4.2). Enriched results are summarized in common data table formats using the R packages tidyverse (v2.0.0) and reactable (v0.4.4). In cases where no significant results could be produced given the user input and analysis configuration, a pop-up window is triggered to inform the user and suggest alternative approaches.

## 3 Implementation

**Figure 1.**
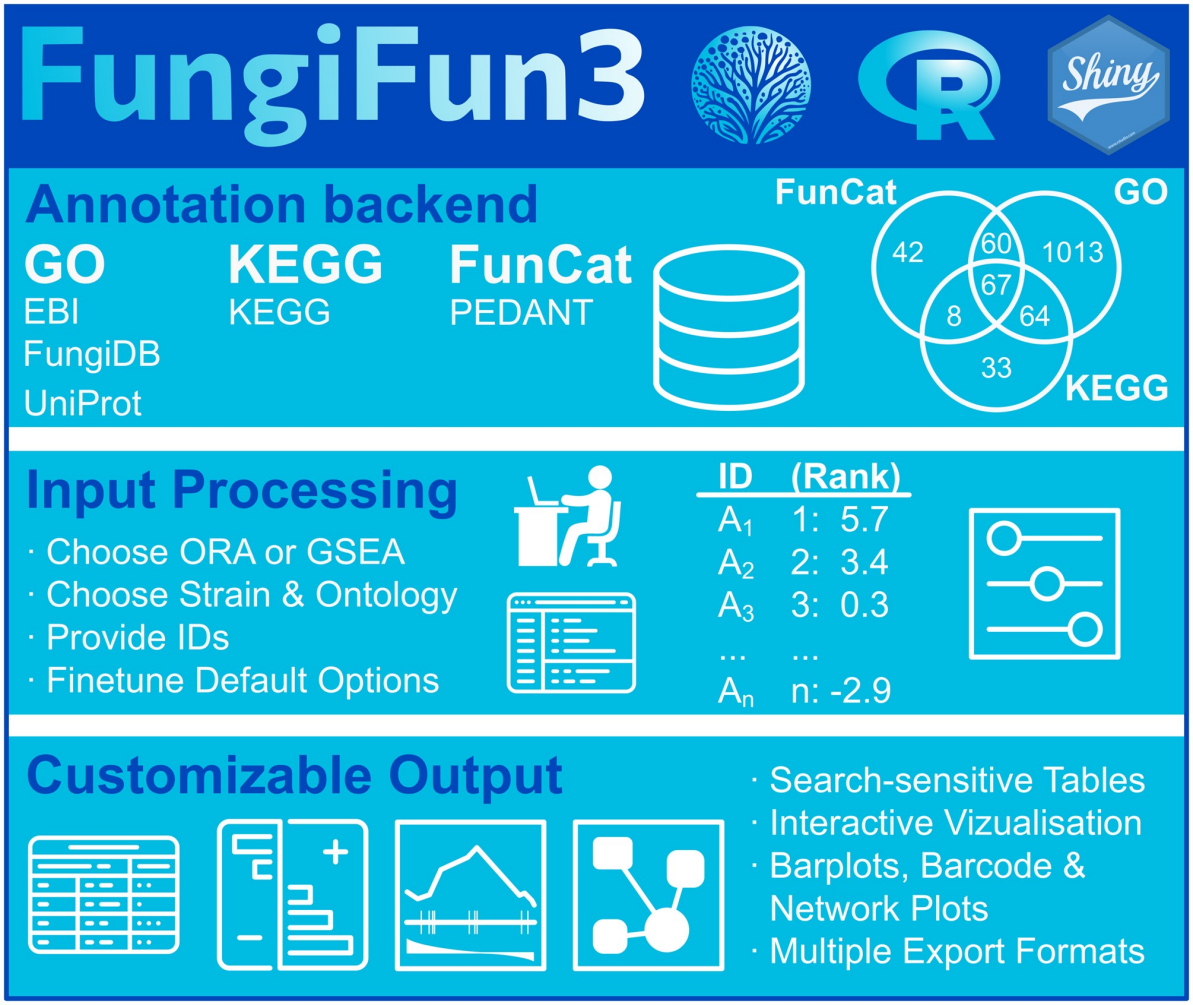
FungiFun3 overview. The backend MySQL database includes annotations for gene-to-category information from multiple resources for three ontologies [Gene Ontology [GO], KEGG and FunCat (Legacy support)]. Upon choosing overrepresentation analysis (ORA) the user decides, which strain to analyze and provides supported strain IDs (enables ORA analysis). Examples of supported IDs are provided for each strain. Optionally, rank information, e.g. by fold-changes between two compared conditions can be provided (enabling gene set enrichment analysis—GSEA). After optionally fine-tuning default parameters, significance tests and output objects are generated, which can be searched, modified, and downloaded in different formats.

For gene set analysis FungiFun3 supports two analysis modes: ORA requiring an unranked list of gene IDs as input (one item per row), or GSEA requiring a ranked two-column table as input (with one ID and rank value, e.g. fold change or statistical *P*-value, per row, Fig. 1).

Upon choosing ORA and after selecting the strain of interest from a search-sensitive entry list, the available gene set resource for enrichment—GO, KEGG, or FunCat—for the specified strain can be chosen. For each resource and strain, the supported gene IDs are provided. An “Advanced settings” panel allows fine-tuning standard parameters, including the choice of the background database (whole database or custom list of IDs), the accepted significance threshold and multiple test correction method, the exact significance test to perform, and the annotation type (directly or also indirectly, to include higher ranked annotated categories in the GO scheme). The resulting enriched categories can then be interrogated online via an all-column sensitive search bar in an interactive table. Overview plots summarize the portion of significantly enriched categories among all input-associated categories as well as the portion of genes associated with significant categories. Furthermore, the top 10 most significant categories are displayed by default, but can be changed to any enriched category in a searchable drop-down list.

If the user provides ranked input information, i.e. gene lists ranked by fold change between two conditions of interest, GSEA can be performed taking this information into account. Of note, to prevent biased results, for GSEA all gene rank information regardless of significance status has to be provided as input. Multiple visualizations including ranked barcode and dot plots, network graphs and upset plots summarize the detected enriched terms.

All result objects are available for download in different formats, including xlsx, jpg, and pdf.

## 4 Conclusion

We present FungiFun3, a major update of our web application tailored towards gene set enrichment analysis for fungal species. The R shiny framework allowed for modern user input-output management and improved user experience. Without the need for programming, both ORA and GSEA approaches for functional enrichment analysis are freely available for everyone, including non-programmers. The number of supported strains increased substantially and includes all strain annotations across various common databases including EBI, FungiDB, UniProt, and KEGG. The specific focus of FungiFun3 for enrichment analysis enables high maintainability while keeping the complexity of the web application low and thus accessible. Thus, regular updates of the knowledge database as well as ease-of-use of the web application are guaranteed and will contribute to enhanced interrogation of fungal molecular functions to reveal e.g. pathogenicity or antifungal resistance mechanisms for a full variety of fungal species.

## Supplementary Material

btae620_Supplementary_Data

## Data Availability

The annotation information underlying this article were derived from sources in the public domain: gene ontology (https://release.geneontology.org/), FungiDB (https://fungidb.org/common/downloads/Current_Release/), and EBI (ftp://ftp.ebi.ac.uk/pub/databases/GO/goa/proteomes). The Kegg pathway annotations were retrieved via REST API: https://rest.kegg.jp/link/pathway/. The FunCat resource is supported as LEGACY support, since its original resource is offline (http://pedant.gsf.de). Further data on supported annotations is available in the online supplementary material.
